# Exploring access to social protection by persons with disabilities in Bangladesh

**DOI:** 10.1371/journal.pone.0321887

**Published:** 2025-04-16

**Authors:** Mizanur Rahman, Md Shohel Rana, Md Mostafizur Rahman, Md Nuruzzaman Khan

**Affiliations:** 1 Jamalpur Science and Technology University, Jamalpur, Bangladesh; 2 Department of Population Science, Jatiya Kabi Kazi Nazrul Islam University, Mymensingh, Bangladesh; 3 Maternal and Child Health Division, International Centre for Diarrhoeal Disease Research, Bangladesh (icddr,b), 68 Shaheed Tajuddin Ahmed Sarani, Mohakhali, Dhaka, Bangladesh; 4 Department of Population Science and Human Resource Development, University of Rajshahi, Bangladesh; 5 Nossal Institute for Global Health, Melbourne School of Population and Global Health, The University of Melbourne, Melbourne, Australia; BRAC University, BANGLADESH

## Abstract

**Background:**

Social protection programs have played a significant role in ensuring that persons with disabilities (PWD) in low- and middle-income countries have access to basic livelihoods. However, there is a lack of research examining the extent of social protection program coverage for PWD and the factors influencing their inclusion. This study aimed to explore the extent of PWD’s inclusion in social protection programs in Bangladesh and the factors influencing their inclusion in these programs.

**Methods:**

We analyzed data from the 2021 National Household Survey on Persons with Disabilities in Bangladesh. The outcome variable considered was PWD’s inclusion in social protection programs (yes, no) to receive support, as well as the timing of inclusion in the social protection program (non-inclusion (0), inclusion within 0–6 months of the survey (1), and inclusion more than 6 months before the survey (2)). Explanatory variables included factors at the individual, household, and community levels. A multilevel multinomial logistic regression model was used to explore the associations between the outcome variable and explanatory variables, with respondents categorized into two groups based on age (children (0 to <18) and adults and older (18–95 years)).

**Results:**

Data from a total of 4,293 PWD were analyzed, with a mean age of 41.4 years; 59% of the respondents were male. Approximately 37.7% (95% CI, 36.0–39.6) of the total respondents reported inclusion in social protection programs within 0–6 months of the survey, rising to 47.4% (95% CI, 45.6–49.2) for support received more than 6 months before the survey. “Disability allowances (69.0%) were the most common type of social protection program that PWD reported being included in, followed by old age allowances (16.3%) and assistance through the VGD/VGF programs (6.8%). Among children aged <18 years, the likelihood of inclusion in social protection programs was higher for those with multiple disabilities. In contrast, for PWD aged 18 years and older, inclusion in social protection programs was lower among those with mental illness, hearing disabilities, and intellectual disabilities. The likelihood of inclusion in social protection programs was higher for older, unmarried, widowed, divorced, or separated PWD. Conversely, PWD from wealthier households and those residing in the Dhaka division had a lower likelihood of being included in social protection programs.

**Conclusion:**

The findings of this study underscore the urgent need for more comprehensive and inclusive social protection policies and programs to support the well-being of PWD in Bangladesh. Since disability grants are the primary source of social protection for this group, it is crucial to expand coverage and increase the amount of financial support provided.

## Introduction

Social protection programs, such as old age allowances and widow allowances, are commonly implemented in low- and middle-income countries (LMICs) by both government and non-government organizations (NGOs) [1]. These initiatives aim to provide protection for individuals and households facing specific needs due to poverty and other vulnerabilities, with the ultimate goal of enhancing their livelihoods [2,3]. By doing so, the overarching objectives are to meet the basic needs of vulnerable populations, encompassing aspects such as health and education, while integrating them into mainstream society [2]. Predominantly, social protection programs in LMICs take the form of financial assistance or the provision of food, distributed to beneficiaries at fixed intervals, often on a monthly basis [4,5]. Several research studies conducted across diverse settings have demonstrated the effectiveness of these social protection programs in improving the livelihoods of vulnerable populations [6–8]. Consequently, these programs have been incorporated as a major target within the Sustainable Development Goals (SDGs, target 1.3) to be achieved by 2030, with the aim of reducing and preventing poverty and enhancing the livelihoods of vulnerable populations [9].

With 16% of the global population, persons with disabilities (PWD) constitute the world’s largest minority group [10]. Over 80% of them reside in LMICs, with their numbers steadily increasing due to factors such as improved survival rates among individuals who born with disabilities and the rise in disabilities resulting from road traffic injuries and other causes [10,11]. Notably, a significant proportion of this population resides within the lower socio-economic segments, indicating that the majority of PWD, especially people of younger ages, are living in poverty [12–14]. Moreover, their limited income-generating opportunities render them highly reliant on social protection programs [15,16]. The prevalence of predominant socio-cultural norms in LMICs, which often attribute disabilities to curses and perceive them as permanent burdens, further exacerbates their situation [17,18]. This situation is particularly pronounced in Bangladesh [7,19], where approximately 3% of the total population is classified as PWD, and such misconceptions are widespread.

However, despite these challenges and the substantial number of PWD in Bangladesh, it remains unclear what percentage of them are covered by existing social protection programs and what specific forms of support they receive [20,21]. Furthermore, the types and varieties of social protection programs available for PWD remain undocumented [19]. The limited estimates provided by the Ministry of Social Welfare of Bangladesh do not align with the true prevalence of PWD and solely account for the support they offer [22]. NGOs also play a role in implementing social protection programs; however, their coverage remains undisclosed [23]. Additionally, the factors associated with inclusion in social protection programs are not well-understood in Bangladesh, as like other LMICs, as existing studies have reported conflicting associations due to small sample sizes and less precise data analysis methods [24,25]. This study aims to address these gaps by examining the inclusion of PWDs in social protection programs for social support in Bangladesh and identifying the factors that influence their inclusion in these programs.

## Methods

### Sampling strategy

Data were extracted from the 2021 National Survey on Persons with Disabilities (NSPD) conducted by the Bangladesh Bureau of Statistics (BBS) [26]. The survey employed a two-stage stratified random sampling technique to identify the nationally representative households from where respondents were included. In the first stage of sampling, 800 primary sampling units (PSUs) were selected randomly from the list of 293,579 PSUs generated by the Bangladesh Bureau of Statistics as part of the 2011 National Population Census. Household listing operation was then conducted in each selected PSU. Subsequently, 45 households were systematically chosen from each selected PSU in the second of sampling. This approach yielded a roster of 36,000 households, covering urban-rural and city- corporation areas from each of the eight divisions of Bangladesh. Of them data collection were undertaken in 35,493 households, attaining a coverage rate of 98.6%. There were 155,025 respondents in these selected households and all of them were included in the survey. Detailed description regarding the survey has been presented elsewhere [26].

### Analytical sample

**Fig 1:**
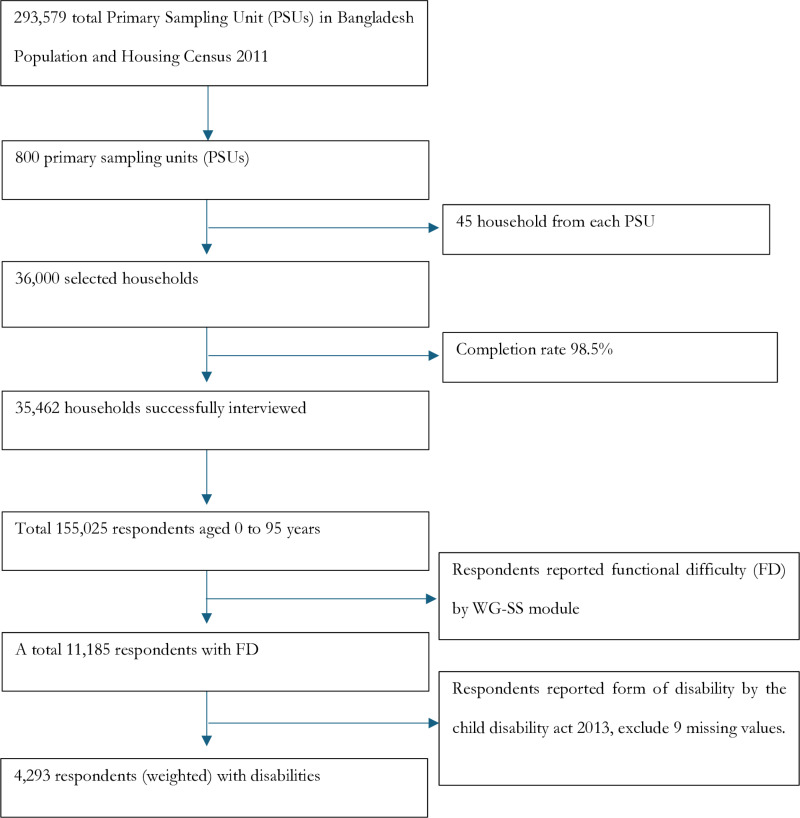
Sample selection procedure to analyzed in this study.

We analyzed data from 4,293 respondents, which represents 2.79% of the total NSPD sample (Fig 1). The criteria for inclusion were: (i) persons with self-reported disability and (ii) respond to the questions related to social protection. The survey collected disability-related data using the Washington Group on Disability guidelines [26,27], which align with the 2013 Bangladesh Act on the Rights and Protection of Persons with Disabilities [28,29]. According to these guidelines, disabilities for children and adults were recorded across six domains: seeing, hearing, walking, remembering, self-care, and communication.

### Outcome variables

The outcome variable was respondents’ inclusion in any social protection program, categorized as ‘yes’ or ‘no.’ Respondents were asked, “*Have you or (name) ever received any help from this type of program?*” The response options were ‘yes’ (indicating participation and receipt of support through the listed programs) and ‘no’ (indicating non-participation and non-receipt of support). A list of social protection programs administered by both government and non-government organizations was provided, along with an option to write in any support received that was not listed. These programs included disability allowances, education stipends for PWD, family allowances for freedom fighters, old age allowances, widow allowances, maternity allowances, assistance for vulnerable group development (VGD), vulnerable group feeding (VGF), and other related allowances.

Initially, this was used as the outcome variable. However, when exploring factors associated with social protection program inclusion, we divided the outcome variable into three categories based on responses to an additional question about timing. If respondents answered ‘yes’ to the first question, they were asked, “*If so, how many months ago did you (or name) last receive this assistance?*” Respondents could specify how many months prior they had received social protection services, and relevant documents (e.g., VGF cards) were checked when applicable. Based on these responses, we created a variable with three categories: non-inclusion (0, reported ‘no’ to the first question), inclusion in social support programs within 0–6 months of the survey (1, reported ‘yes’ to the first question and within 0–6 months of the second question), and inclusion in social support program more than 6 months before the survey (2, reported ‘yes’ to the first question and more than 6 months before the second question). This segregation was necessary to enable a more detailed analysis of the timing of inclusion in social support programs and to examine how the predictors of recent and past support differ.

### Explanatory variables

We used a comprehensive two-stage selection process to select explanatory variables. We first generate a list of potential explanatory variables based on the extensive review of the available relevant studies conducted in LMICs [24,30–32]. Subsequently, these identified variables were cross-referenced with the survey data to verify their availability. Those variables that were available in the survey were subsequently classified according to the socio-ecological model of health into three tiers: individual level factors, households level factors, and community level factors. The individual-level factors encompassed the respondent’s age (children aged 0-<18 years, adults aged 18–59 years and older aged 60 and above), years of schooling (treated as continuous variable), gender (male or female), occupation (agriculture, blue-collar work, pink-collar work, white-collar work, student, housewife, and others), marital status (married, unmarried, or widowed/divorced/separated) and religion (Islam, others). Wealth quintiles of households (poorer, poorest, middle, richer, richest) was included as the household-level variable. This variable was formulated by the survey authority by using principal component analysis of household asset-related variables, such as roofing type and ownership of a refrigerator. Respondents’ place of residence (urban and rural) and region of residence (Barishal, Chattogram, Dhaka, Khulna, Mymensingh, Rajshahi, Rangpur, and Sylhet) were considered as community level variables.

### Statistical analysis

Descriptive statistics were used to explore the characteristics of the respondents. We analyzed access to social protection among PWD using descriptive statistics, considering factors such as age, gender, type of disability, assistance received, and other relevant variables. The Chi-square test was used to examine the statistical significance of variations in social protection program inclusion across the considered explanatory variables.

To explore the likelihood of inclusion in social protection programs by disability type, we ran multilevel logistic regression models, treating participation in social protection programs (yes/no) as the outcome variable and disability types as the main explanatory variable. Other individual, household, and community-level factors were included as covariates. A multilevel multinomial logistic regression model was used to explore factors associated with timing of inclusion in social support programs to receive social support, categorized as non-inclusion (0), inclusion in social in social support program within 0–6 months (1), and inclusion in social support program more than 6 months before the survey (2).

For both cases, two separate models were run, splitting the total sample into two groups based on age: 0–<18 years (children) and 18–95 (adult and older aged). This division was made due to the varying coverage of social protection programs for inclusion by children and adults. The use of multilevel modelling in both cases was necessary because the 2021 NSPD employed a two-stage stratified random sampling technique based on a nested source of variability, where individuals were nested within households, and households were nested within clusters [33–35]. To account for this structure, multilevel modelling with a random intercept at both the household and cluster levels was used. Previous research has shown that multilevel modelling provides more precise results for hierarchical data than conventional logistic regression models. We also compared fixed-effects multinomial modelling with random-effects (multilevel) multinomial modelling using a likelihood ratio test. The significant results further supported the need for multilevel modelling. Multicollinearity was checked before running each model, and if evidence of multicollinearity was found, the relevant variable was removed, and the model was rerun. Results are reported as adjusted Risk Ratios (aRR) or adjusted Odds Ratios (aOR) with their corresponding 95% Confidence Intervals (95% CI). The complex survey design was accounted for in all analyses using Stata’s “svy” command. All statistical analyses were conducted using STATA/SE 14.0 (Stata Corp LP, College Station, Texas, United States).

### Ethics approval and consent to participate

The survey data we analyzed was ethically reviewed and approved by the Ethics Committee of the Bangladesh Bureau of Statistics (BBS). We obtained de-identified data from the BBS after submitting a research proposal outlining our adherence to the Helsinki Declaration. Informed written consent was obtained before starting the interviews. Since the data is anonymized, further ethical approval for this specific study was not required.

## Result

### Background characteristics of the respondents

The background characteristics of the respondents are presented in Table 1. The study included a total of 4,293 respondents, where around 59% of the respondents were male. The average age of the respondents was 41.4 years, with more than half of the total respondents being 18–59 years old. However, around one third of total respondents identified them as unable to work. Over 80% of the total respondents resided in rural area while around 22% indicated Dhaka as their region of residence.

**Table 1:** Background characteristics of the persons with disability in Bangladesh, 2021, n=4,293.

Characteristics	Frequency (n=4,293)	Percentage (95% CI)
Respondent’s age, mean (SD)		41.4 (±23.6)
Children aged 0-<18 year	892	20.8 (19.51–22.1)
Adults aged 18–59 year	2,190	51.0 (49.5–52.5)
Older aged 60 and above	1,211	28.2 (26.7–29.7)
Gender		
Male	2,514	58.5 (57.0–60.1)
Female	1,779	41.5 (40.0–43.0)
Respondents’ year of schooling, mean (SD)		3.9(±9.0)
Respondent’s occupation		
Agriculture	411	9.6 (8.7–10.6)
Blue collar worker ^b^	290	6.8 (6.0–7.6)
Pink collar worker ^P^	164	3.8 (3.3–4.5)
White collar worker ^w^	376	8.7 (7.9–9.7)
Student	484	11.3 (10.3–12.3)
Housewives	505	11.8 (10.8–12.8)
Unable to work	1,413	32.9 (31.4–34.5)
Others^*^	650	15.2 (14.0–16.4)
Marital Status		
Married	2,062	48.0 (46.5–49.6)
Unmarried	1,505	35.1 (33.4–36.7)
Widow/Divorce/Separate	726	16.9 (15.8–18.1)
Religion		
Muslim	3,843	89.5 (87.4–91.3)
Hindu and others^**^	450	10.5 (8.7–12.6)
Wealth Quintile		
Poorest	1,164	27.1 (25.2-29.1)
Poorer	942	22.0 (20.5-23.5)
Middle	853	19.9 (18.5-21.4)
Richer	726	16.9 (15.5-18.5)
Richest	607	14.1 (12.7-15.7)
Place of residence		
Rural	3,470	80.8 (79.4-82.2)
Urban	823	19.2 (17.8-20.6)
Administrative division		
Barishal	227	5.3 (4.6-6.0)
Chattogram	697	16.2 (15.0-17.6)
Dhaka	923	21.5 (20.1-23.0)
Khulna	597	13.9 (12.6-15.3)
Mymensingh	311	7.2 (6.5-8.1)
Rajshahi	662	15.4 (14.0-17.0)
Rangpur	633	14.7 (13.4-16.2)
Sylhet	243	5.7 (5.0-6.4)

Note: All the values in table 1 are presented with column percentage.

^b^blue collar worker means [factory/manufacturing workers/labour, transportation/ communication workers, day labor (non-agriculture), auto/ cng/ tempo driver, rickshaw driver/ van driver/ boatman, poultry/ animal husbandry for business, fishery or aquaculture and fisherman]

^p^Pink collar worker means [small business (capital up to taka 1000), business (capital over taka 10000), kabiraj/ojha/spiritual physician, village doctor and homeopathy doctor]

^w^White collar worker means [teacher, lawyer/journalist/doctor/engineer, government employee/officer, private/Ngo employee/ officer, handicraft/cottage industry, weaver/blacksmith/potter/goldsmith/service, imam/ priest, family helper and housemaid/servant]

* family helper, servant, looking for work, unable for work, beggar, no work and not looking for work, and other unnamed occupations;

** Hindu, Buddhism, Christianity, etc.

### Inclusion of persons with disabilities in different social protection programs

Table 2 presents the distribution of inclusion in different types of social protection programs within 0–6 months of the survey and more than 6 months before the survey, through which PWD have received continuous social support. The average participation rate in any social protection program within 0–6 months of the survey was 37.7%, while inclusion in support programs more than 6 months before the survey was 47.4%. The majority of PWD who reported inclusion in social protection programs within 0–6 months of the survey received disability allowances (69%), followed by old age allowances (16.3%) and VGD/VGF (6.8%). A similar pattern emerged among respondents who reported inclusion in social protection programs more than 6 months before the survey. Roughly 60% of eligible PWD, specifically those entitled to the freedom fighters’ allowance, reported not being included in such programs. Similarly, approximately 57% of eligible PWD who were eligible for the educational stipend did not receive it.

**Table 2:** Distribution of inclusion in social protection programs for all persons with disabilities and across eligibility status for specific social protection programs in Bangladesh.

Social protection program types	Total persons with disabilities		Distribution of social support uptake based on eligibility status^+^						
Inclusion in social protection program within 0–6 months of the survey (n=1,620)	Inclusion in social protection program more than 6 months before the survey (n=2,035)	Eligible respondents	Non-inclusion in social support program (n=2,258)		Inclusion in social protection program within 0–6 months of the survey (n=1,620)		Inclusion in social protection program more than 6 months before the survey (n=415)	
% (95% CI)	% (95% CI)	n	% (n)	95% CI	% (n)	95% CI	% (n)	95% CI
Disability allowances	69.0 (66.4–71.6)	64.1 (61.7–66.5)	4,293	52.6 (2,258)	50.7–54.4	37.8 (1,620)	36.0–39.6	9.6 (415)	8.6–10.7
Education stipend for disabled persons	1.0 (0.6–1.7)	2.3 (1.7–3.0)	427	57.1 (244)	52.3–61.8	30.9 (132)	26.8–35.4	12.0 (51)	9.1–15.5
Freedom freighter family allowances	0.9 (0.5–1.6)	0.7 (0.4–1.3)	682	60.0 (409)	56.0–63.8	30.8 (210)	27.1–34.8	9.2 (63)	7.2–11.7
Old age allowances	16.3 (14.4–18.3)	15.3 (13. 7–17.0)	1,362	48.3 (657)	45.2–51.3	42.1 (574)	39.2–45.1	9.6 (131)	8.1–11.5
Widow allowances	3.8 (3.0–4.9)	3.7 (2.9–4.7)	714	47.5 (339)	43.5–51.5	41.9 (299)	38.1–45.8	10.6 (76)	8.5–13.4
Maternity allowance	–	0.1 (0.0–0.40)	774	55.4 (429)	51.6–59.1	34.8 (269)	31.3–38.5	9.8 (76)	7.8–12.3
Assistance–VGD/VGF	6.8 (5.5–8.5)	9.0 (7.7–10.5)	4,292	52.6 (2,258)	50.8–54.5	37.7 (1,619)	35.9–39.5	9.7 (415)	8.7–10.7
Money/Food for work	1.1 (0.7–1.8)	2.7 (2.0–3.5)	2,916	50.8 (1,480)	48.6–53.0	39.1 (1,140)	37.0–41.3	10.1 (296)	8.9–11.5
Others allowance	1.1 (0.6–1.7)	2.2 (1.6–3.1)	3,933	51.5 (2,029)	49.7–53.5	38.5 (1,512)	36.6–40.3	10.0 (392)	8.9–11.1
Overall allowance acceptance	37.7 (36.0–39.6)	47.7 (45.6–49.2)							

**Note:** We assessed eligibility based on the background characteristics of the respondents. For example, in the case of the freedom fighter family allowance, we examined the total number of eligible persons with disabilities among families qualified to receive the freedom fighter allowance and how many of them reported receiving this allowance; ^+^Presented values are in row percentage.

### Reasons for non-inclusion in social support programs among children aged 0–<18

**Fig 2:**
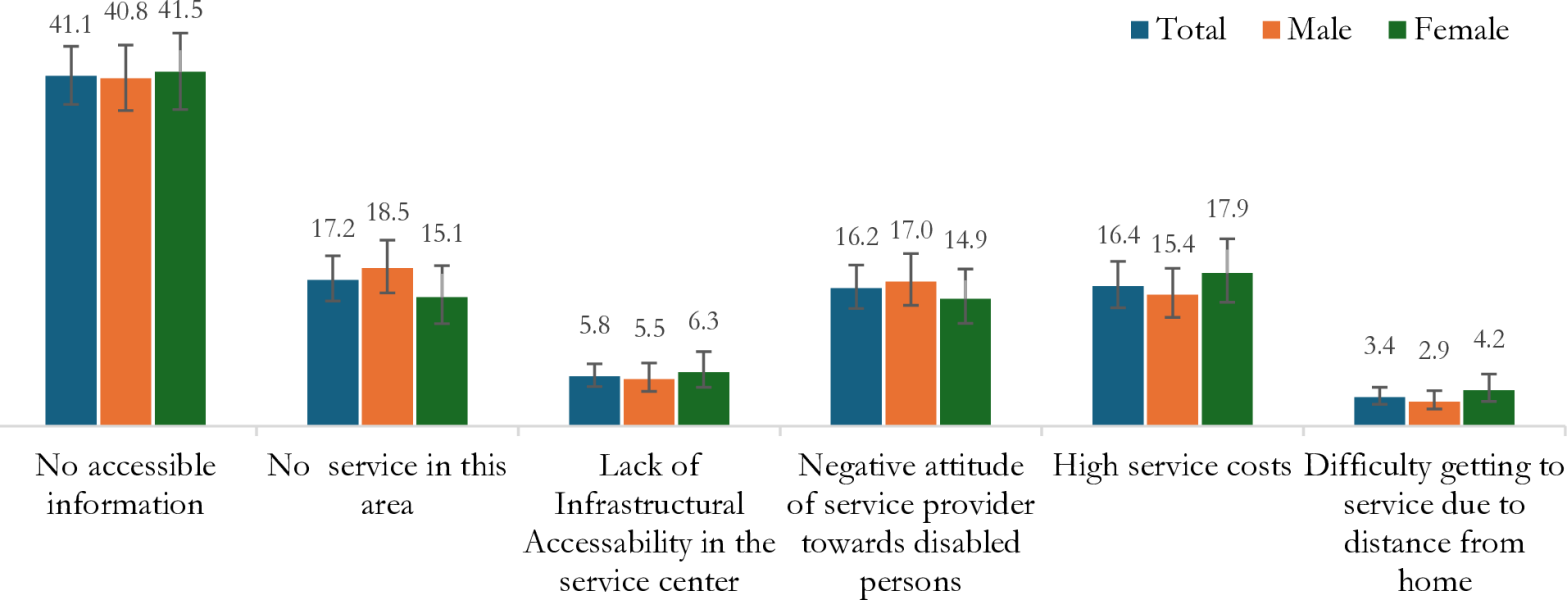
Reasons for not inclusion in social protection programs for children aged <18 years with disability in Bangladesh.

We also explored the reasons for non-inclusion in social support programs for children aged 0-<18 among those who reported not being included in any social support programs (Fig 2). The most common reason identified was a lack of accessible information regarding the availability of services (41.0%), followed by the unavailability of services in the residing area (17.2%), high service costs (16.4%), and negative attitudes towards services (16.2%). Gender differences were observed in the reasons for not accessing social support. Data for adults aged 18 or older did not provide information on their reasons for non-inclusion in social protection programs, so we were unable to report on this matter.

### Distribution of social protection program inclusion for persons with disabilities across individual, household, and community factors

Table 3 presents distribution of PWD’s inclusion in social protection program across considered individual, household and community level factors. Persons with disabilities who aged 60 or older, was unable to work and who classified as widowed, divorced, or separated reported higher percentage of inclusion in social protection program. At the regional level, it was found that PWD who residing in the Mymensingh and Barishal divisions reported higher inclusion in social protection programs. We found statistically significant variations of inclusion in social protection program across considered individual, household, and community-level characteristics, except respondents’ gender and religion.

**Table 3:** Inclusion in social protection programs of persons with disability at anytime (n=4293).

Characteristics	Inclusion in Social Protection Programs		P–value^+^
	Yes	No	
**Individual level factor**			p<0.001
**Respondent’s age**			
Children aged 0–<18 year	39.2 (350)	60.8 (542)	
Adults aged 18–59 year	47.7 (1,043)	52.4 (1,146)	
Older aged 60 and above	52.9 (641)	47.1 (570)	
**Gender**			p=0.278
Male	46.7 (1,174)	53.3 (1340)	
Female	48.4 (861)	51.6 (918)	
**Respondent’s year of schooling**	2.9 (2,070)	4.9 (2,132)	p<0.001
**Respondent’s occupation**			p<0.001
Agriculture	47.5 (195)	52.6 (216)	
Blue collar worker	42.2 (122)	57.8 (168)	
Pink collar worker	44.6 (73)	55.5 (91)	
White collar worker	51.4 (193)	48.6 (183)	
Student	41.6 (201)	58.4 (282)	
Housewives	40.7 (205)	59.3 (299)	
Unable to work	55.0 (777)	45.0 (636)	
Others	41.1 (267)	58.9 (383)	
**Marital Status**			p<0.001
Married	44.8 (923)	55.2 (1,139)	
Unmarried	48.0 (723)	52.0 (782)	
Widow/Divorce/Separate	53.5 (388)	46.5 (338)	
**Household level factor**			
**Religion**			p=0.066
Muslim	46.8 (1,800)	53.2 (2,043)	
Hindu and others	52.2 (235)	47.8 (215)	
**Wealth Quintile**			p<0.001
Poorest	52.5 (611)	47.6 (554)	
Poorer	53.1 (500)	46.9 (442)	
Middle	47.5 (405)	52.5 (448)	
Richer	43.3 (315)	56.7 (412)	
Richest	33.6 (204)	66.4 (403)	
**Community level factor**			
**Place of residence**			p<0.001
Rural	49.4 (1,713)	50.6 (1,757)	
Urban	39.0 (321)	61.0 (502)	
**Administrative division**			p<0.05
Barishal	51.9 (118)	48.1 (109)	
Chattogram	46.7 (325)	53.3 (371)	
Dhaka	41.5 (383)	58.5 (540)	
Khulna	48.2 (288)	51.8 (310)	
Mymensingh	54.5 (170)	45.5 (141)	
Rajshahi	50.5 (335)	49.5 (328)	
Rangpur	46.7 (295)	53.3 (337)	
Sylhet	50.0 (122)	50.0 (122)	

Note: All values in table 3 are presented with row percentage, ^+^p–value obtained from the chi– square test except variable respondent’s year of schooling where p-value obtained from proportion test.

### Likelihood of inclusion in social protection program by disability types

We ran two adjusted multilevel logistic regression models, stratifying the total sample into two sub-groups: children aged 0 to <18 years and adults and older aged 18 years or more (Table 4), to explore the likelihood of inclusion in social support programs by disability type, adjusted for covariates. We found divergent patterns in the inclusion in social protection programs for PWD aged 0-<18 years and those aged 18 years or more. Among PWD aged 0-<18 years, the likelihood was higher for those with multiple co-occurring disabilities (aOR = 2.2, 95% CI: 1.0–4.9) compared to those with Autism/Autism Spectrum Disorder. In contrast, for PWD aged 18 and more, lower likelihoods of inclusion in social protection programs were reported for those with mental illness (aOR = 0.1, 95% CI: 0.0–0.3), hearing disabilities (aOR = 0.1, 95% CI: 0.0–0.5), and intellectual disabilities (aOR = 0.1, 95% CI: 0.0–0.6), compared to those with autism or autism spectrum disorder.

**Table 4:** Multilevel logistic regression models estimating the adjusted likelihood of receiving social support allowance by disability type.

Type of disabilities	Total (N=4,293)	Children (aged 0–<18 years) with disabilities for whom inclusion in a social protection program was reported at any time before the survey (ref: non-inclusion in a social protection program)	Adults and older (aged 18–95 years) with disabilities for whom inclusion in a social protection program was reported at any time before the survey (ref: non-inclusion in a social protection program)
n (%)	aOR (95% CI)	aOR (95% CI)
Autism/Autism spectrum disorder	69 (1.6)	1.0	1.0
Physical disability	1,822 (42.5)	0.7 (0.3–1.5)	0.3 (0.1–1.1)
Mental illness disability	360 (8.4)	0.4 (0.2–1.2)	0.1 (0.0–0.3) ^***^
Visual disability	606 (14.1)	0.6 (0.2–1.4)	0.3 (0.1–1.2)
Speech disability	175 (4.1)	1.0 (0.4–2.4)	0.3 (0.1–1.2)
Intellectual disability	211 (4.9)	0.6 (0.3–1.4)	0.1 (0.0–0.6) ^**^
Hearing disability	299 (7.0)	0.4 (0.1–1.5)	0.1 (0.0–0.5) ^***^
Hearing–Visual disability	20 (0.5)	–	0.2 (0.0–1.0) ^**^
Cerebral Palsy	119 (2.7)	2.0 (0.8–5.0)	0.8 (0.1–4.5)
Down syndrome	52 (1.2)	0.5 (0.1–1.6)	0.2 (0.0–1.0)
Multidimensional disability	498 (11.6)	2.2 (1.0–4.9) ^**^	0.4 (0.1–1.4)
Others	62 (1.4)	0.1 (0.0–0.6) ^***^	0.1 (0.0–0.6) ^**^
Cluster–level variance (SE)		0.54 (0.30)	0.66 (0.07)
ICC		8%	12%
AIC		1002.0	4404.9
BIC		1132.4	4632.0

**Notes:** Models are adjusted for the individual, households and community level factors described in the explanatory variable section.

** = p<0.05,

*** = p<0.01

### Individual, household, and community characteristics associated with social protection program inclusion for persons with disabilities

We also two separate multilevel multinomial logistic regression models (for individuals aged 0 to <18 years and those aged 18 years or older) to explore individual, households and community level factors associated with inclusion in social protection program (Table 5).

**Table 5:** Multilevel multinomial mixed effect logistic regression model assessing factors associated with inclusion in social protection program by the persons with disabilities in Bangladesh.

Characteristics	Children aged 0–<18		Adult and older aged 18–95	
	Inclusion in social protection program within 0–6 months of the survey aRR (95% CI)	Inclusion in social protection program more than 6 months before the survey aRR (95% CI)	Inclusion in social protection program within 0–6 months of the survey aRR (95% CI)	Inclusion in social protection program more than 6 months before the survey aRR (95% CI)
**Individual level factor**				
**Respondent’s age**	1.1 (1.1–1.2) ^***^	1.1 (1.1–1.2) ^***^	1.0 (1.0–1.0) ^**^	1.0 (1.0–1.0)
**Gender**				
Male	1.0	1.0	1.0	1.0
Female	1.9 (1.3–2.6) ^***^	1.6 (1.0–2.7)	0.8 (0.6–0.9) ^**^	1.1 (0.8–1.6)
**Respondent’s year of schooling**	0.9(0.9–1.0) ^**^	1.0(1.0–1.0)	0.9 (0.9–1.0) ^***^	0.9(0.9–1.0) ^**^
**Respondent’s occupation**				
Agriculture	na	na	1.0	1.0
Blue collar worker	na	na	0.9 (0.6–1.3)	1.1 (0.7–1.9)
Pink collar worker	na	na	1.2 (0.8–1.7)	1.4 (0.7–2.5)
White collar worker	na	na	1.5 (1.1–2.1) ^**^	1.0 (0.5–1.6)
Student	na	na	1.7 (0.9–2.9)	1.9 (0.8–4.5)
Housewives	na	na	1.1 (0.8–1.6)	0.8 (0.5–1.5)
Unable to work	na	na	1.5 (1.1–1.9) ^***^	1.0 (0.6–1.5)
Others	na	na	1.2 (0.9–1.7)	1.6 (1.0–2.5)
**Marital Status**				
Married	na	na	1.0	1.0
Unmarried	na	na	2.5 (1.9–3.2) ^***^	1.6 (1.0–2.4) ^**^
Widow/Divorce/Separate	na	na	1.4 (1.1–1.7) ^***^	1.0 (0.7–1.4)
**Household level factor**				
**Religion**				
Muslim	1.0	1.0	1.0	1.0
Hindu and others	0.8 (0.4–1.5)	0.9 (0.3–2.4)	1.2 (1.0–1.5)	1.4 (0.9–2.0)
**Wealth Quintile**				
Poorest	1.0	1.0	1.0	1.0
Poorer	1.4 (0.9–2.1)	1.3 (0.6–2.7)	1.0 (0.8–1.2)	1.0 (0.7–1.4)
Middle	0.9 (0.5–1.4)	0.6 (0.2–1.4)	0.8 (0.6–1.0) ^**^	0.9 (0.6–1.3)
Richer	1.0 (0.6–1.7)	1.2 (0.5–2.8)	0.6 (0.5–0.8) ^***^	0.6 (0.4–1.0) ^**^
Richest	0.9 (0.5–1.7)	2.2 (0.9–5.2)	0.4 (0.3–0.6) ^***^	0.3 (0.2–0.6) ^***^
**Community level factor**				
**Place of residence**				
Rural	1.0	1.0	1.0	1.0
Urban	0.9 (0.6–1.5)	1.1 (0.5–2.3)	0.9 (0.7–1.1)	1.1 (0.8–1.6)
**Administrative division**				
Barishal	1.0	1.0	1.0	1.0
Chattogram	1.0 (0.5–2.4)	1.4 (0.4–5.3)	0.9 (0.6–1.2)	0.7 (0.4–1.3)
Dhaka	0.9 (0.4–2.0)	1.2 (0.3–4.5)	0.6 (0.4–0.9) ^***^	0.9 (0.5–1.7)
Khulna	1.3 (0.6–3.1)	0.6 (0.1v2.8)	0.9 (0.6–1.4)	0.7 (0.4–1.3)
Mymensingh	1.0 (0.4–2.4)	0.8 (0.1–3.8)	1.1 (0.7–1.7)	1.0 (0.5–2.0)
Rajshahi	1.3 (0.6–3.0)	0.9 (0.2–3.6)	0.9 (0.6–1.3)	0.9 (0.5–1.6)
Rangpur	1.0 (0.5–2.4)	1.0 (0.3–4.2)	0.8 (0.6–1.2)	0.7 (0.4–1.3)
Sylhet	0.9 (0.3–2.3)	2.2 (0.5–9.4)	0.8 (0.5–1.3)	1.3 (0.7–2.6)

Note:

** =p<0.05,

*** =p<0.01, na= Not applicable

In the model for PWD aged 0 to <18 years, a one-year increase in age was associated with a 10% (95% CI: 1.1–1.2) higher likelihood of inclusion in a social protection program within 0–6 months of the survey and more than 6 months before the survey. Furthermore, females with disabilities reported 1.9 times (95% CI, 1.3–2.6) and 1.6 times (95% CI, 1.0–2.7) higher likelihoods of inclusion in social protection programs compared to males with disabilities.

In the model for PWD aged 18 years and older, we found females reported 20% (aRR = 0.8, 95% CI: 0.6–0.9) lower likelihoods of inclusion in social protection programs within 0–6 months of the survey compared to their males counterparts. Persons with disabilities working in white-collar jobs (aRR = 1.5, 95% CI: 1.1–2.1) and those unable to work (aRR = 1.5, 95% CI: 1.1–1.9) had higher likelihoods of inclusion in social protection programs within 0–6 months of the compared to those who engaged in agricultural work. Compared to married individuals, the likelihood of inclusion in social protection programs within 0–6 months of the survey was 2.5 times (95% CI: 1.9–3.2) higher among unmarried PWD and 1.4 times (95% CI: 1.1–1.7) higher among widowed, divorced, or separated PWD. This relationship remained significant only for unmarried individuals (aRR, 1.6, 95% CI, 1.0–2.4) when examining inclusion in social protection program more than 6 months before the survey. Compared to PWD residing in poorer households, the likelihood of inclusion in social protection programs decreased as household wealth quintile increased. Furthermore, PWD residing in the Dhaka division had a 40% (aRR = 0.6, 95% CI: 0.4–0.9) lower likelihood of inclusion in social protection programs within 0–6 months of the survey compared to those residing in the Barishal division.

## Discussion

The study had two primary objectives: first, to assess the extent of inclusion of PWD in social protection programs in Bangladesh, and second, to identify the factors associated with PWD’s inclusion in these programs. Only 38% of PWD in Bangladesh reported being enrolled in any social protection programs within 0–6 months of the survey, a figure that increased to 48% when including those who had been enrolled more than 6 months before the survey. Disability allowances (69.0%) were the most prevalent type of social protection program, followed by old age allowances (16.3%) and assistance within the VGD/VGF programs (6.8%). For PWD aged 18 years and older, inclusion in social protection programs was lower among those with mental illness, hearing disabilities, and intellectual disabilities. Older PWD and individuals who were unmarried, widowed, divorced, or separated reported higher rates of inclusion in social protection programs. Conversely, PWD residing in more affluent households and in the Dhaka division were less likely to be included in social protection programs. For PWD aged 0-<18 years, the likelihood of inclusion in social protection programs was higher if they had multiple forms of disability. Increasing age and being female were linked with a higher likelihood of being involved in social protection programs within 0–6 months of the survey. These findings highlight the inadequacy of social protection program inclusion for persons with disabilities, with nearly two-thirds excluded from any program within 0–6 months of the survey.

This study highlights that social protection programs in Bangladesh include only 38% of PWD within 0–6 months of the survey, a figure that increases to 48% in case of inclusion in social protection program more than 6 months before the survey. Within this group who received support within 0–6 months of the survey, 69% reported inclusion in disability allowances tailored for them, while the remaining reported inclusion in other program and received assistance, such as old age or widowed allowances. These findings underscore that a majority of PWD in Bangladesh remain excluded from social protection programs. Even among those included, roughly one-third are not integrated into programs designed to address their specific needs. This situation is significantly worse than in Pakistan (63%) and Vietnam (83.5%), where social protection coverage for PWD is much higher, although it aligns with the Maldives (42.7%) and exceeds that of India (21%) [36–38]. The lower coverage of social protection program in Bangladesh is attributed to the government’s lower allocation of resources for vulnerable populations compared to the demand, despite an overall increase in the budget over the years [22]. For example, in the most recent 2023–2024 national budget in Bangladesh, only BDT 2978.71 core (2.8 billion USD) is allocated for PWD, with a continuous monthly allowance of only 850 BDT (7.73 USD) [22]. Along with this lower funding availability, in many cases, this amount does not reach the intended recipients, with consistent evidence of incorrect beneficiary selection based on political identity, personal relationships with local leaders, and corruption among local leaders [6,39]. This issue is compounded by poor governance, administrative complexity, and a lack of effective monitoring, further emphasizing the challenging conditions faced by PWD in Bangladesh [6,40]. This indicates the challenging circumstances faced by PWD in Bangladesh, with rising costs that many PWD cannot afford, including increasing demand for healthcare services and regular visits to healthcare centers, which are often located in cities [8,41]. Consequently, a significant proportion of PWD (22–25%) struggle to meet their basic needs, including food and clothing [1,20].

Complexity also arises in national-level policies and programs that are designed to provide social support [7]. For instance, according to current policies and programs, children with disabilities are only eligible for a disability allowance if they meet specific criteria: (1) they are aged six years or older, (2) they are resided in a family with an annual income of less than 36,000 BDT (360 USD), and (3) they are diagnosed with particular type of disabilities [42]. However, with rapid economic development, finding someone in Bangladesh with an annual income less than 36000 BDT has become increasingly unlikely. Furthermore, restricting eligibility to only a specific type of children with disabilities aged six years or older automatically excludes a significant number of children with disabilities [43]. A similar challenge exists for educational stipends, as children with disabilities are ineligible unless they are enrolled in government-recognized educational institutions [18,44]. This is often not the case, as they may attend schools not recognized by the government, like many children with disabilities who enroll in Madrasah education, which is often not government registered [5,44]. An additional challenge is the prioritization of female children, as found in this study, which sometimes results in male children being left behind due to this focus [6,15].

For adult PWD, we found a decreased likelihood of inclusion in social protection programs among female persons with disabilities and people with higher levels of education and wealth quintiles, as reported in other studies in LMICs [30,31,41]. Several factors could potentially contribute to these findings. For females with disabilities, cultural and societal factors may come into play. Gender biases and disparities in opportunities and decision-making could affect their access to social protection programs [45,46]. They also often face multiple layers of marginalization, due to both their gender and status of disabilities, making them more vulnerable and potentially less likely to access social protection programs [2,45]. Moreover, women with disabilities may contend with additional caregiving responsibilities, which can limit their ability to engage with these programs. Regarding persons with higher levels of education and wealth quintiles, there may be a perception that they are more self-sufficient and less in need of social protection support [43,47]. This assumption might lead to their exclusion from these programs, even though they may still require assistance [13,41]. Similarly, people in higher wealth quintiles may have greater financial resources to address their needs independently, reducing their reliance on social protection programs [6,14]. Furthermore, these disparities in inclusion may also result from variations in awareness and outreach efforts, with marginalized groups potentially receiving less information about available support [15,44]. Lastly, administrative complexities and issues with program accessibility may also contribute to these disparities, as persons with greater access to information and resources may navigate these challenges more effectively [10,19].

Conversely, this study found that older PWD, those with white-collar occupations, those unable to work, and individuals who were unmarried, widowed, or divorced/separated had higher probabilities of inclusion in social protection programs. These findings are consistent with research from other LMICs [4,30,32,48]. Several factors may underline these findings. As age of PWD increased, they may face greater challenges in terms of their physical or economic well-being, making them more vulnerable and increasing the likelihood of their inclusion in social protection programs [19]. Moreover, with the increasing age, they may be able to negotiate with the local leaders or program implementers to include them in the programs, otherwise they are mostly unidentified [7,30]. Persons with disabilities in white-collar occupations may have a stronger voice in advocating for their rights to be included in social protection programs, due to a better understanding of their eligibility and how to navigate the system [14]. In comparison, PWD who are unable to work may face particularly acute financial hardships, prompting social protection programs to be more responsive to their needs and more inclined to include them [21,41]. Similarly, unmarried, widowed, or divorced/separated PWD may be seen as more vulnerable or in need of additional support, leading social protection programs to be more likely to encompass them. This increased inclusion could be driven by a combination of legal and policy considerations, which explicitly target these individuals as eligible beneficiaries, and a lack of alternative support systems for this demographic, making social protection programs a critical source of assistance [49]. Additionally, gender disparities and the societal recognition of their vulnerabilities may further contribute to their prioritization in such programs [50]. These insights highlight the complex interplay of individual characteristics, socioeconomic factors, and program design, emphasizing the need for tailored policies that address the unique needs and circumstances of PWD [25,51]. These findings are also consistent with research from other LMICs and suggest that certain demographic and socioeconomic factors play a role in determining who is included in social protection programs [4,25].

The findings of this study have important policy implications, particularly regarding the need for immediate reforms to enhance the inclusion of PWD in social protection programs in Bangladesh, where only 38% report enrolment. Policymakers should expand eligibility criteria to ensure broader access, especially for children and individuals from diverse socioeconomic backgrounds, including women and the elderly. Addressing systemic barriers is crucial, which involves improving governance, reducing corruption, and simplifying administrative processes. Tailored outreach campaigns should specifically target marginalized groups, particularly women and people in higher wealth quintiles, to raise awareness of available support. Furthermore, programs must be designed with consideration of the unique challenges faced by different demographic groups to ensure that all PWD can effectively access the necessary support.

This study has several strengths and a few limitations. To the best of our knowledge, this is the first study in Bangladesh that explored the support received by PWD under the social support program at the national level and its correlates. It is based on a quite large sample collected through a nationally representative household survey, and recognized procedures were applied to measure social protection. Data were analyzed using comprehensive statistical modeling with a hierarchical structure of the data, and sampling weights were considered in all analyses. Therefore, the findings are robust and can be used in developing national-level policies and programs. However, the primary limitations of this study include the analysis of cross-sectional data, which limited our capacity to establish causality, and the findings were fully correlational. Data were collected through questions posed to the respondents with no opportunity for validation, indicating the possibility of recall bias, although any such bias is likely to be random. Moreover, collecting information from PWD affecting mental functioning is challenging, which can lead to misreporting within this group. While we included a category for multidimensional disability, where individuals with more than one form of disability were accounted for, this inclusion was not based on medical examination. It is possible that some PWD affecting multiple domains reported only one. Furthermore, aside from the factors adjusted in the model, health and environmental variables can contribute to the onset of disability, making them important to be considered in the model. However, these data were not available in the survey, limiting our ability to do so. Nonetheless, despite these limitations, the findings of this study will contribute to national-level policy and program development.

## Conclusions

These findings highlight the inadequate coverage of social protection programs for PWD. Policymakers should expand coverage of social protection services for PWD, particularly for children, women, and the elderly. Additionally, both the coverage and the amount of financial support provided through disability allowance grants need to be prioritized for expansion. Future research should be conducted to address the limitations of this study and to better understand the coverage of social protection programs in Bangladesh and the impacts of their absence.

## List of Abbreviations

VGD: Vulnerable Group Development
VGF: Vulnerable Group Feeding
PWD: Persons with disability
WHO: World Health Organization
SBI: Short Birth Interval
LMICs: Low– and middle–income countries
BBS: Bangladesh Bureau of Statistics;
aRR: Adjusted Relative Risk
aOR: , Adjusted Odds Ratios
AIC: Akaike Information Criteria
BIC: Bayesian Information Criteria

## References

[1] World Bank TWBSP, Labor Strategy. Resilience, Equity, and Opportunity. World Bank Washington, DC; 2012.

[2] ILO. World social protection report 2017-19: universal social protection to achieve the sustainable development goals: International Labour Organisation (ILO); 2017.

[3] Gentilini U, Omamo SW. Social protection 2.0: Exploring issues, evidence and debates in a globalizing world. Food Policy. 2011;36(3):329–40. doi: 10.1016/j.foodpol.2011.03.007

[4] The Informational Basis of Emerging Social Assistance in Low and Middle-Income Countries. GDI Working Paper 2018-023. Manchester: The University of Manchester. [Internet]. 2018-23.

[5] Wang D, Fawzi WW. Impacts of school feeding on educational and health outcomes of school-age children and adolescents in low- and middle-income countries: protocol for a systematic review and meta-analysis. Syst Rev. 2020;9(1):55. doi: 10.1186/s13643-020-01317-6 32178734 PMC7075040

[6] Masud-All-Kamal M, Saha C. Targeting social policy and poverty reduction: The case of social safety nets in Bangladesh. Poverty Public Policy. 2014;6(2):195–211.

[7] Kidd S. Social exclusion and access to social protection schemes. J Develop Effective. 2017;9(2):212–44. doi: 10.1080/19439342.2017.1305982

[8] Mizunoya S, Mitra S. Is there a disability gap in employment rates in developing countries?. World Develop. 2013;42:28–43. doi: 10.1016/j.worlddev.2012.05.037

[9] Hák T, Janoušková S, Moldan B. Sustainable Development Goals: A need for relevant indicators. Ecol Indic. 2016;60:565–73. doi: 10.1016/j.ecolind.2015.08.003

[10] World Health Organization (WHO). Global report on health equity for persons with disabilities. Geneva: World Health Organization; 2022. Licence: CC BY-NC-SA 3.0 IGO. 2022.

[11] Haghani M, Behnood A, Dixit V, Oviedo-Trespalacios O. Road safety research in the context of low- and middle-income countries: Macro-scale literature analyses, trends, knowledge gaps and challenges. Safety Science. 2022;146:105513. doi: 10.1016/j.ssci.2021.105513

[12] Mitra S, Palmer M, Kim H, Mont D, Groce N. Extra costs of living with a disability: A review and agenda for research. Disabil Health J. 2017;10(4):475–84. doi: 10.1016/j.dhjo.2017.04.007 28501322

[13] Braithwaite J, Mont D. Disability and poverty: A survey of world bank poverty assessments and implications. Alter. 2009;3(3):219–32. doi: 10.1016/j.alter.2008.10.002

[14] Palmer M, Groce N, Mont D, Nguyen OH, Mitra S. The economic lives of people with disabilities in Vietnam. PLoS One. 2015;10(7):e0133623. doi: 10.1371/journal.pone.0133623 26197034 PMC4510056

[15] Mitra S, Posarac A, Vick B. Disability and poverty in developing countries: a Multidimensional Study. World Develop. 2013;41:1–18. doi: 10.1016/j.worlddev.2012.05.024

[16] Hanass-Hancock J, McKenzie TC. People with disabilities and income-related social protection measures in South Africa: Where is the gap?. Afr J Disabil. 2017;6:300. doi: 10.4102/ajod.v6i0.300 29062759 PMC5645570

[17] Zheng Q, Tian Q, Hao C, Gu J, Tao J, Liang Z, et al. Comparison of attitudes toward disability and people with disability among caregivers, the public, and people with disability: findings from a cross-sectional survey. BMC Public Health. 2016;16(1):1024. doi: 10.1186/s12889-016-3670-0 27686163 PMC5043610

[18] UNICEF. The Role of Social Norms in Decisions to Provide Schooling to Children with Disabilities in East and Southern Africa. 2021.

[19] Hussain MMM. Social exclusion of people with disability in Bangladesh: dimensions and challenges. ASWJ. 2021;6(1):12–21. doi: 10.47405/aswj.v6i1.161

[20] Sustainable economic empowerment of persons with disabilities [Internet]. Aug 23, 2021.

[21] Shamsul Alam TFE. Challenges in implementing social protection programmes. Apr 2017.

[22] Ministry of Social Welfare. Ministry of Social Welfares’ related information to be included in the Economic Review-2020. Economic Review. 2020;2020.

[23] Hassan ABME. NGOs and their implications in promoting social development in Bangladesh: an overview. Soc Anthrop. 2015;3(1):24–36. doi: 10.13189/sa.2015.030104

[24] Bernabe-Ortiz A, Diez-Canseco F, Vasquez A, Kuper H, Walsham M, Blanchet K. Inclusion of persons with disabilities in systems of social protection: a population-based survey and case–control study in Peru. BMJ Open. 2016;6(8):1–9. 10.1136/bmjopen-2016-011300 PMC5013477 27566630

[25] Nuri RP, Ghahari S, Aldersey HM, Huque AS. Exploring access to government-led support for children with disabilities in Bangladesh. PLoS One. 2020;15(7):e0235439. doi: 10.1371/journal.pone.0235439 32614867 PMC7332059

[26] Bangladesh Bureau of Statistics (BBS) SD, M.o.P.,. National Survey on Persons with Disabilities (NSPD) 2021. Bangladesh Bureau of Statistics. Bangladesh Bureau of Statistics, 2022.

[27] Rahman M, Rana M, Khandaker G, Rahman M, Khan Mnj. National burden of disability in Bangladesh and its socio-demographic correlates. medRxiv. 2023;2023:23295500. doi: 10.1101/2023.09.13.23295500

[28] Nuri RP, Aldersey HM, Ghahari S, Huque AS, Shabnam J. The Bangladeshi rights and protection of persons with disability act of 2013: a policy analysis. J Disabil Policy Stud. 2022;33(3):178–87. doi: 10.1177/10442073211066789 36397763 PMC9650720

[29] Madans JH, Loeb ME, Altman BM. Measuring disability and monitoring the UN Convention on the rights of persons with disabilities: the work of the washington group on disability statistics. BMC Public Health. 2011;11 Suppl 4(Suppl 4):S4. doi: 10.1186/1471-2458-11-S4-S4 21624190 PMC3104217

[30] Banks L, Walsham M, Neupane S, Neupane S, Pradhananga Y, Maharjan M. Access to social protection among people with disabilities: Mixed methods research from Tanahun, Nepal. European J Develop Res. 2019;31:929–56.

[31] Walsham M, Kuper H, Banks LM, Blanchet K. Social protection for people with disabilities in Africa and Asia: a review of programmes for low- and middle-income countries. Oxford Development Studies. 2018;47(1):97–112. doi: 10.1080/13600818.2018.1515903

[32] Banks LM, Kuper H, Polack S. Poverty and disability in low- and middle-income countries: A systematic review. PLoS One. 2017;12(12):e0189996. doi: 10.1371/journal.pone.0189996 29267388 PMC5739437

[33] Peugh JL. A practical guide to multilevel modeling. J Sch Psychol. 2010;48(1):85–112. doi: 10.1016/j.jsp.2009.09.002 20006989

[34] Rabe-Hesketh S, Skrondal A. Multilevel modelling of complex survey data. J Royal Stat Soc Series A: Stat Soc. 2006;169(4):805–27. doi: 10.1111/j.1467-985x.2006.00426.x

[35] Twisk J. Applied multilevel analysis: a practical guide for medical researchers. 2006.

[36] ADB BRIEFS. Disability and Social Protection in Asia. 2021.

[37] Lorraine Wapling RSaDS. Social Protection and Disability in India. February 2021.

[38] Office. IL. World Social Protection Report 2020–22:. Social Protection at the Crossroads – in Pursuit of a Better Future. Geneva: ILO, 2021.

[39] Devereux S, White P. Social protection in Africa: evidence, politics and rights. Poverty Public Policy. 2010;2(3):53–77. doi: 10.2202/1944-2858.1078

[40] Schurmann AT, Mahmud S. Civil society, health, and social exclusion in Bangladesh. J Health Popul Nutr. 2009;27(4):536–44. doi: 10.3329/jhpn.v27i4.3400 19761087 PMC2928100

[41] Jahan N, Holloway C. Barriers to access and retain formal employment for persons with disabilities in Bangladesh and Kenya. GDI Hub Working Paper Series, 2020.

[42] Al Imam MH, Jahan I, Das MC, Muhit M, Akbar D, Badawi N, et al. Situation analysis of rehabilitation services for persons with disabilities in Bangladesh: identifying service gaps and scopes for improvement. Disabil Rehabil. 2022;44(19):5571–84. doi: 10.1080/09638288.2021.1939799 34176400

[43] Maloni P, Despres E, Habbous J, Primmer A, Slatten J, Gibson B, et al. Perceptions of disability among mothers of children with disability in Bangladesh: Implications for rehabilitation service delivery. Disabil Rehabil. 2010;32(10):845–54.doi: 10.1080/09638288.2023.1234567 20131951

[44] Hayes AM, Bulat J. Disabilities inclusive education systems and policies guide for low-and middle-income countries. RTI International. 2017. 32125793

[45] Pradhan MAH, Afrin S. A review of social safety nets programs for women in Bangladesh: issue and challenges. aeb. 2015;3(4):149–56. doi: 10.13189/aeb.2015.030405

[46] Oridi FI, Uddin MdS, Faisal-E-Alam Md, Husain T. Prevailing factors of rural women entrepreneurship in Bangladesh: evidence from handicraft business. J Glob Entrepr Res. 2022;12(1):305–18. doi: 10.1007/s40497-022-00327-z

[47] Beblavý M, Thum A-E, Veselkova M. Education and social protection policies in OECD countries: Social stratification and policy intervention. Journal of European Social Policy. 2013;23(5):487–503. doi: 10.1177/0958928713499174

[48] Organization. WH. World report on disability 2011.2011:1–300.

[49] Devandas Aguilar C. Social protection and persons with disabilities. Int Social Security Review. 2017;70(4):45–65. doi: 10.1111/issr.12152

[50] Perera C, Bakrania S, Ipince A, Nesbitt‐Ahmed Z, Obasola O, Richardson D. Impact of social protection on gender equality in low‐and middle‐income countries: A systematic review of reviews. European J Develop Res. 2022;18(2):e1240. 10.1002/cl2.1240 PMC9133545 36913187

[51] Park E-Y, Kim J-H. Interaction of socio-demographic characteristics on acceptance of disability among individuals with physical disabilities. Front Psychiatry. 2021;12:597817. doi: 10.3389/fpsyt.2021.597817 33995137 PMC8113681

